# The Inhibition of Bromodomain and Extraterminal Domain (BET) Proteins Protects Against Microglia-Mediated Neuronal Loss In Vitro

**DOI:** 10.3390/biom15040528

**Published:** 2025-04-04

**Authors:** Marta Matuszewska, Anna Wilkaniec, Magdalena Cieślik, Marcin Strawski, Grzegorz A. Czapski

**Affiliations:** 1 Mossakowski Medical Research Institute, Polish Academy of Sciences, Department of Cellular Signalling, ul. Pawińskiego 5, 02-106 Warsaw, Poland; mmatuszewska@imdik.pan.pl (M.M.); awilkaniec@imdik.pan.pl (A.W.); mcieslik@imdik.pan.pl (M.C.); 2 University of Warsaw, Faculty of Chemistry, ul. Pasteura 1, 02-093 Warsaw, Poland; marcin@chem.uw.edu.pl

**Keywords:** neuroinflammation, bromodomain and extraterminal domain proteins, neuroprotection, Alzheimer’s disease, microglia

## Abstract

Neuroinflammation is a key feature of all neurodegenerative disorders, including Alzheimer’s disease, and is tightly regulated by epigenetic mechanisms. Among them, bromodomain and extraterminal domain (BET) proteins play a crucial role by recognizing acetylated histones and acting as transcriptional co-regulators to modulate gene expression. This study investigates the potential of inhibiting BET proteins in preventing microglia-mediated neuronal damage in vitro. Murine BV2 microglial cells were exposed to lipopolysaccharide (LPS) or amyloid-β (Aβ) to induce an inflammatory response, and the subsequent effects on murine HT22 neuronal cells were examined. Among the BET proteins tested, only Brd4 was significantly upregulated in BV2 cells upon pro-inflammatory stimulation. JQ1, a potent pan-inhibitor of BET proteins, suppressed LPS-induced upregulation of pro-inflammatory cytokine mRNA levels, including *Il1b*, *Il6*, and *Tnf*, in BV2 microglia. Pre-treatment with JQ1 attenuated the cytotoxicity of LPS-activated BV2 cells toward neurons. Additionally, conditioned media from Aβ fibril-stimulated BV2 cells induced neuronal cell death, which was partially prevented by pre-treatment with JQ1. Co-culture assays further demonstrated the beneficial effect of BET inhibition. Our findings suggest that targeting BET proteins may offer a neuroprotective strategy by modulating microglial activation, potentially providing therapeutic benefits in neurodegenerative diseases.

## 1. Introduction

The immune system identifies and eliminates pathogens, as well as recognizes and neutralizes abnormal self-molecules, thereby contributing to the maintenance of homeostasis within the organism. However, when dysregulated, prolonged, or excessive, immune responses can contribute to the development of pathological conditions. This is particularly evident in the central nervous system (CNS), where neuroinflammation is a central feature of a wide range of neurodegenerative diseases, including Alzheimer’s disease (AD), Parkinson’s disease (PD), and multiple sclerosis [1,2,3]. In these disorders, chronic neuroinflammation exacerbates neuronal damage and contributes to disease progression [4,5,6]. This process is primarily driven by activated microglia, the brain’s resident immune cells, which are essential for maintaining homeostasis and responding to injury or disease. In their surveillance state, often inaccurately referred to as the “resting state”, microglial cells monitor the CNS environment and maintain neuronal health by clearing cellular debris and secreting neurotrophic factors. However, microglia become activated in response to pathological stimuli, such as amyloid-β (Aβ) peptides in AD or α-synuclein in PD [7,8]. This activation releases pro-inflammatory cytokines, reactive oxygen species (ROS), and neurotoxic molecules, impairing neuronal function and promoting neurodegeneration [9,10]. Therefore, the modulation of microglial activation presents a potential therapeutic target for preventing or delaying the progression of neurodegenerative diseases.

Bromodomain and extraterminal domain (BET) proteins, including Brd2, Brd3, and Brd4, have emerged as key regulators of transcriptional processes [11]. These proteins function as “readers” of the histone acetylation code, recognizing acetylated lysine residues on histones and other proteins, which promotes the recruitment of transcriptional complexes and modulates gene expression [12]. BET proteins play a crucial role in various cellular processes, including inflammation, cell cycle regulation, and differentiation. Studies have shown that BET inhibitors, such as JQ1, can attenuate inflammatory signaling pathways in macrophages by blocking the transcription of pro-inflammatory genes, including TNF-α, IL-6, and IL-1β [13]. However, while the role of BET proteins in macrophage-mediated inflammation is well-established, their involvement in microglial activation and neuroinflammation is still less defined.

Given the growing evidence linking neuroinflammation to the development of neurodegenerative diseases, this study investigates the therapeutic potential of inhibiting BET proteins as a neuroprotective strategy. In this in vitro model, we specifically investigate whether inhibition of BET proteins can prevent microglia-dependent neuronal cell loss. Using the murine BV2 microglial cell line and HT22 neuronal cells, we assess the impact of BET inhibition on microglial activation and the subsequent effects on neuronal viability. This work aims to provide further insights into the role of BET proteins in neuroinflammation and their potential as therapeutic targets in neurodegenerative diseases.

## 2. Materials and Methods

### 2.1. Reagents and Materials

(S)-(+)-tert-butyl 2-(4-(4-chlorophenyl)-2,3,9-trimethyl-6H-thieno[3,2-f][1,2,4]triazolo [4,3-a][1,4]diazepin-6-yl)acetate (JQ1), L-glutamine, penicillin, streptomycin, 3-(4,5-dimethyl-2-tiazolilo)-2,5-diphenyl-2H-tetrazolium bromide (MTT), DNase I, anhydrous DMSO, lipopolysaccharide (LPS) from *Escherichia coli* O55:B5 (toxicity 3,000,000 U/mg), hexafluoroisopropanol (HFIP), bovine serum albumin (BSA), and accutase solution were from Merck Millipore Inc. (Burlington, MA, USA). RPMI-1640 medium, fetal bovine serum (FBS), phosphate-buffered saline (PBS), Nunc Polycarbonate Cell Culture Inserts in Multi-Well Plates, pore size 0.4 µm, TRI reagent, High-Capacity cDNA Reverse Transcription Kit (including RNase Inhibitor), TaqMan Gene Expression Assays and TaqMan Fast Advanced Master Mix, red fluorescent microspheres (FMS; 2.0 μm), and BCA Protein Assay Kits were from Thermo Fisher Scientific, Inc. (Waltham, MA, USA). Ham’s F12 Medium, w/o L-glutamine and phenol red, was from PAN-Biotech GmbH (Aidenbach, Germany). Amyloid-beta (Aβ1-42) was from AnaSpec, Inc. (Fremont, CA, USA). The enzyme-linked immunosorbent assay (ELISA) kits for quantitatively detecting mouse Brd2, Brd3, and Brd4 proteins were from Abbexa Ltd. (Cambridge, UK). The Mycoplasma Detection Kit was from InvivoGen Corp. (San Diego, CA, USA). Mycoplasma-Off disinfecting solution was from Minerva Biolabs GmbH (Berlin, Germany). The Mini Dialysis Kit (1 kDa cut-off) was from Cytiva (Buckinghamshire, UK). All other reagents were obtained from Merck Millipore Inc.

### 2.2. Reagents and Treatments

Bacterial LPS was dissolved in PBS and added to a cell culture medium to induce the pro-inflammatory activation of BV2 microglial cells. The cells were treated with LPS at a concentration of 100 ng/mL for the specified durations [14,15]. HFIP-pretreated Aβ1–42 was initially dissolved in anhydrous DMSO at a concentration of 5 mM, followed by dilution in F12 medium to a final concentration of 100 µM. The solution was vortexed for 30 s and then incubated under different conditions to promote the formation of distinct aggregated species: (1) for oligomer formation, the solution was incubated at 4 °C for 24 h; (2) for protofibrils, it was incubated at 37 °C for 6 h, and (3) for fibrils, incubation was continued at 37 °C for 60 h [16,17]. After incubation, the samples were subjected to dialysis in F12 for 3 h using a Mini Dialysis Kit to remove any residual solvents or small molecules [16]. BET inhibitor JQ1 was initially dissolved in DMSO to create a 10 mM stock solution, which was subsequently diluted in the culture medium to achieve a 50 µM solution for application to the cells [12]. The final concentration in the cell culture medium (50 nM) was selected based on our previous studies [18]. Equal volumes of vehicles were added to each experimental group to ensure comparable conditions.

### 2.3. Cell Culture Experiments

Murine microglial BV2 cells [19] (Elabscience Biotechnology Inc., Houston, TX, USA) and murine neuronal HT22 cells [20] (Merck Millipore Inc., Burlington, MA, USA) were cultured in the same conditions in RPMI-1640 medium supplemented with 10% FBS, 2 mM L-glutamine, 50 units/mL penicillin, and 50 μg/mL streptomycin in a humidified incubator with 5% CO_2_ at 37 °C. The cells were usually passaged every 2 to 3 days, and the passages up to 20 were utilized for the experiments. To exclude mycoplasma contamination, the cells were regularly tested using a Mycoplasma Detection Kit (InvivoGen Corp., San Diego, CA, USA).

A 6 h stimulation period was chosen for the BV2 cells. Our preliminary experiments and published data demonstrate that the expression of most inflammation-related immediate-early genes in microglia increases rapidly and reaches its peak levels within 6 h of stimulation [21,22,23]. Therefore, because BET proteins regulate gene transcription, our study focused on acute processes.

The BV2 cells were pretreated with JQ1 (50 nM) and/or LPS (100 ng/mL) for 6 h for conditioned medium experiments. Subsequently, the conditioned medium was collected and transferred to HT22 neuronal cells. The conditioned medium was mixed with the existing culture medium at a 1:1 ratio [14]. Incubation was continued for 24 h.

For co-culture experiments, we used polycarbonate cell culture inserts in multi-well plates, as previously described [24]. Briefly, BV2 and HT22 cells were seeded and grown separately: BV2 in inserts and HT22 in multi-well plates. After 24 h, the BV2 cells were pretreated with JQ1 (50 nM) and/or after 30 min with Aβ (5 µM) for 6 h. Subsequently, the culture medium was aspirated from the inserts. The inserts containing BV2 cells were then transferred to wells containing HT22 cells and submerged in the resident culture medium. Incubation was continued for 24 h.

### 2.4. MTT Assay

Cell viability was assessed based on the reduction of MTT to formazan [18]. Following treatment with the test compounds, MTT (0.25 mg/mL) was added to the culture medium, and the cells were incubated at 37 °C for an additional 2 h. The medium was gently removed, and the cells were dissolved in DMSO. The absorbance of the formazan formed was measured at 595 nm using a Multiskan GO Microplate Spectrophotometer (Thermo Fisher Scientific, Inc., Waltham, MA, USA).

### 2.5. Gene Expression Analysis

RNA was extracted using a TRI reagent following the manufacturer’s instructions. The RNA concentration and quality were assessed using a Nanodrop 1000 spectrophotometer (Thermo Fisher Scientific, Inc.). DNase I treatment was performed according to the manufacturer’s guidelines to eliminate potential DNA contamination. For reverse transcription, 1 µg of RNA was used as input for cDNA synthesis with the High-Capacity cDNA Reverse Transcription Kit according to the manufacturer’s protocol. Relative mRNA expression levels were determined using the ΔΔCt method, and the results were expressed as relative quantities (RQs) [25]. The levels of mRNA for selected genes were quantified using TaqMan Gene Expression Assays, including *Arg1* (Mm00475988_m1), *Brd2* (Mm01271171_g1), *Brd3* (Mm01326697_m1), *Brd4* (Mm01350417_m1), *Il1b* (Mm00434228_m1), *Il6* (Mm00446190_m1), *Nos2* (Mm00440502_m1), *Tnf* (Mm00443258_m1), and *Gusb* (Mm01197698_m1) [18]. *Gusb* was used as a reference gene. Quantitative PCR was performed on an Applied Biosystems 7500 Real-Time PCR System (Thermo Fisher Scientific, Inc., Waltham, MA, USA) using TaqMan Fast Advanced Master Mix, following the manufacturer’s guidelines.

### 2.6. Enzyme-Linked Immunosorbent Assay (ELISA)

ELISA kits were used strictly in accordance with the manufacturer’s protocols. Briefly, the cells were washed three times with PBS and then homogenized in ice-cold PBS (pH 7.2) using a syringe. Then, the samples were sonicated (40% pulse, 40% power) for 30 s using a Model 150 V/T Ultrasonic Homogenizer (Biologics Inc., Manassas, VA, USA). The sonication was a necessary step [26] to increase the solubility of the BET proteins, which, as chromatin-associated proteins, tend to form insoluble complexes. Then, the homogenates were centrifuged at 10,000× *g* for 5 min, and the supernatant was collected. The protein concentration was accurately determined using a bicinchoninic acid (BCA) assay, with bovine serum albumin (BSA) as the standard. The prepared samples were used immediately to prevent protein degradation or denaturation. After accomplishing all the incubation steps, optical density was measured at 450 nm using a Multiskan GO Microplate Spectrophotometer (Thermo Fisher Scientific, Inc.). The concentration of the tested compound was calculated using a standard curve and normalized to the total protein level. Each sample was analyzed in duplicate.

### 2.7. Phagocytosis Assay

The phagocytic activity of BV2 cells was assessed using flow cytometry. FluoSpheres Carboxylate 2.0 µm fluorescent microspheres (FMSs) were pre-coated with either 3% bovine serum albumin (BSA) for 15 min at 37 °C or 5 µM Aβ for 2 h at 37 °C. The BV2 cells were incubated with 50 µL/mL of FMS for 2 h in standard cell culture conditions. The non-phagocytosed FMSs were removed by washing the cells three times with PBS. The cells were detached using accutase solution and immediately analyzed by flow cytometry (FACSCanto II, BD Biosciences, San Jose, CA, USA) using FACSDiva Software 6.0 (BD Biosciences). The phagocytic index was determined as the percentage of BV2 cells that internalized the FMSs.

### 2.8. Atomic Force Microscopy (AFM)

AFM analysis was performed according to a previously described protocol [22]. Briefly, amyloid samples were prepared by placing a 10 μL drop of the medium onto freshly cleaved mica (V1 grade, NanoAndMore GmbH, Wetzlar, Germany). After a 10 min incubation, each sample was rinsed with deionized water (Merck Millipore Inc., Burlington, MA, USA) and dried using a gentle stream of argon. Imaging of the mica surface and the deposited amyloid structures was performed using PeakForce Tapping^®^ mode on a Multimode 8 Nanoscope atomic force microscope (AFM, Bruker, Billerica, MA, USA). High-resolution probes SNL-10 (Bruker, Billerica, MA, USA) with a spring constant 0.12 Nm^−1^ were used.

### 2.9. Statistics

Statistical analysis was conducted using GraphPad Prism version 8.3.0 (GraphPad Software, San Diego, CA, USA). Comparisons were made using either Student’s *t*-test or one-way analysis of variance (ANOVA) with Bonferroni’s post hoc test for multiple comparisons. Normality was assessed using the Shapiro–Wilk test. The term “n” denotes the number of independent in vitro experiments.

## 3. Results

Considering the crucial role of neuroinflammation in the development of neurodegenerative diseases and the involvement of BET proteins in regulating inflammatory processes, we aimed to elucidate the potential neuroprotective effects of inhibiting BET proteins by examining their influence on microglial activity.

**Figure 1:**
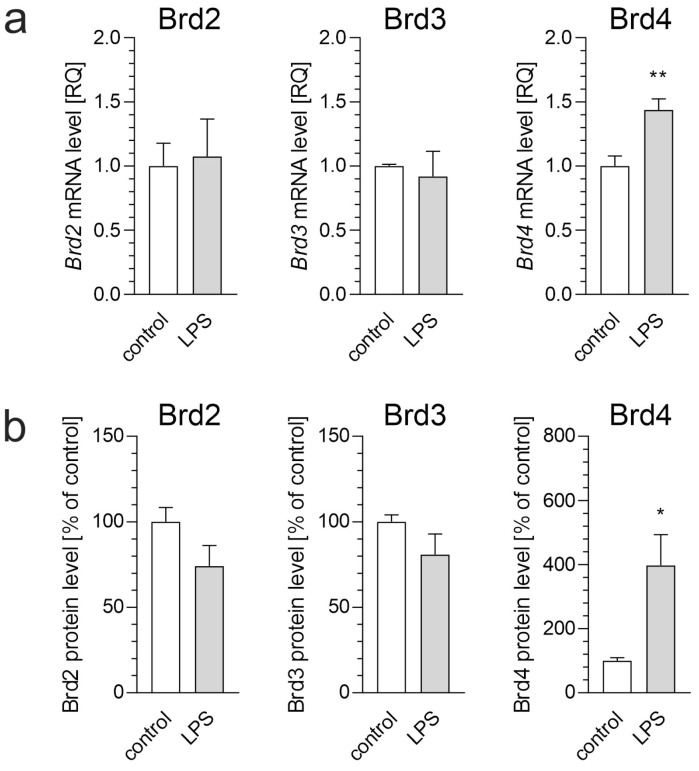
The effect of LPS on BET protein expression in BV2 microglial cells. BV2 cells were treated with LPS (100 ng/mL) for 6 h. (**a**) mRNA levels of *Brd2*, *Brd3*, and *Brd4* were analyzed using qPCR. (**b**) Protein levels of BET proteins were measured using ELISA assays. Data are presented as mean ± SEM; n = 4 (**a**) and 3–4 (**b**); * *p* < 0.05 and ** *p* < 0.01, compared to the control (Student’s *t*-test).

First, we analyzed the impact of LPS on mRNA and protein levels of brain-resident BET family members in BV2 microglial cells. Under control conditions, Brd2, Brd3, and Brd4 showed comparable abundance, measured at 0.46, 0.44, and 0.51 pg/μg of protein, respectively. Our findings demonstrate that Brd4 is the only BET protein significantly upregulated in BV2 microglial cells stimulated with bacterial LPS at 100 ng/mL (Figure 1). This suggests a specific role for Brd4 in microglial activation during neuroinflammation, which is consistent with previous reports implicating BET proteins in regulating inflammatory responses [13].

**Figure 2:**
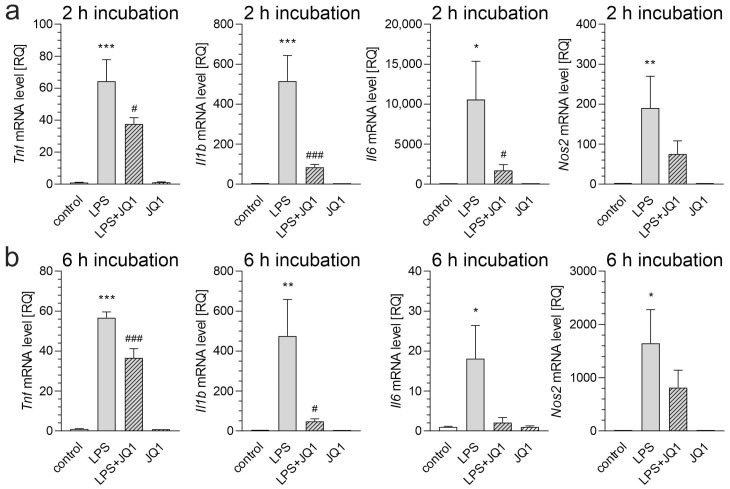
The effect of LPS and/or JQ1 on the mRNA levels of inflammation-related genes in BV2 cells. BV2 cells were treated with JQ1 (50 nM), and after 30 min, LPS (100 ng/mL) was added. Incubation was continued for 2 or 6 h. (**a**) mRNA levels of *Tnf*, *Il1b*, *Il6*, and *Nos2* after 2 h. (**b**) mRNA levels of *Tnf*, *Il1b*, *Il6*, and *Nos2* after 6 h. Data are presented as mean ± SEM; n = 6–9 (**a**) and 6–8 (**b**); * *p* < 0.05, ** *p* < 0.01, and *** *p* < 0.001, compared to the control; # *p* < 0.05 and ### *p* < 0.001, compared to the LPS-treated group (ANOVA with Bonferroni post hoc test).

Treatment with JQ1, a highly specific and potent pan-inhibitor of BET proteins, attenuated the LPS-induced transcription of key pro-inflammatory cytokines, including *Il1b*, *Il6*, and *Tnf*, in the BV2 cells. This indicates that BET inhibition effectively dampens microglial activation, although it does not affect cytokine expression under control conditions (Figure 2).

**Figure 3:**
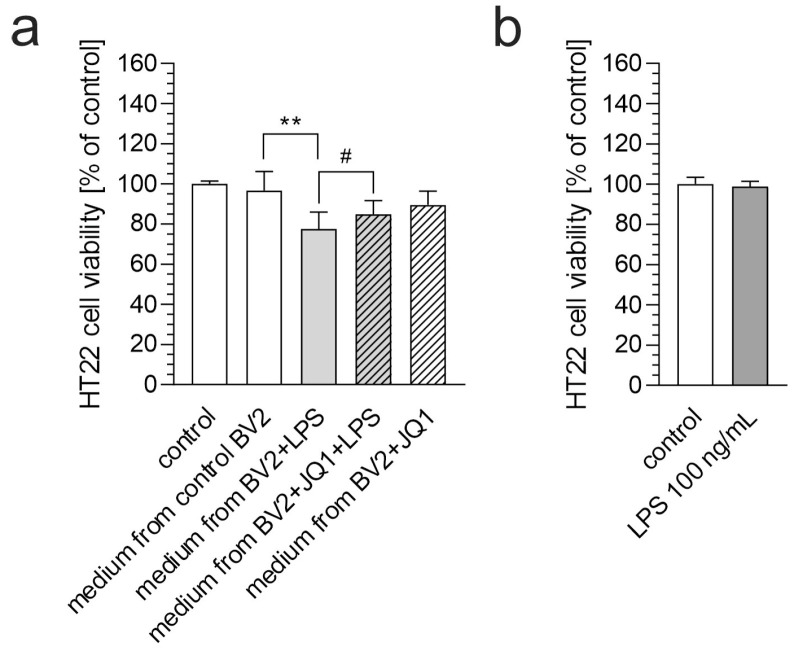
The effect of conditioned medium from LPS- and/or JQ1-treated BV2 cells. (**a**) BV2 cells were treated with JQ1 (50 nM), and then, after 30 min, LPS (100 ng/mL) was added, and incubation was continued for 6 h. The conditioned medium was then transferred to murine neuronal HT22 cells, which were incubated for 24 h. Cell viability was assessed using an MTT assay. (**b**) HT22 cells were treated with LPS (100 ng/mL) for 24 h, and cell viability was evaluated by an MTT assay. Data are presented as mean ± SEM; n = 8 (**a**) and 6 (**b**). ** *p* < 0.01, # *p* < 0.05, compared to the indicated group (ANOVA with Bonferroni post hoc test).

Activated microglial cells produce and release various mediators that may affect neighboring cells. Therefore, we used conditioned media from the LPS-stimulated BV2 cells to study the impact of microglial secretome on neuronal HT22 cells. As shown in Figure 3a, conditioned medium from the control (resting) and JQ1-treated BV2 cells did not affect HT22 cell viability. In contrast, exposure to medium from the LPS-treated BV2 cells induced cell death in the HT22 cells, indicating the neurotoxic effects of inflammation-driven microglial secretions. However, the addition of JQ1 to the BV2 culture prevented LPS-induced microglial activation and subsequent neuronal cell death, significantly preserving the viability of the HT22 cells. These findings underscore the dichotomous role of microglial-derived factors and highlight the therapeutic potential of BET inhibition in counteracting neuroinflammation-mediated toxicity.

To confirm that the cytotoxic effect of the conditioned medium on the HT22 cells was due to microglia-secreted compounds and not residual LPS, we assessed the potential toxicity of LPS at a concentration of 100 ng/mL. As shown in Figure 3b, LPS at this concentration did not affect the viability of the HT22 cells.

**Figure 4:**
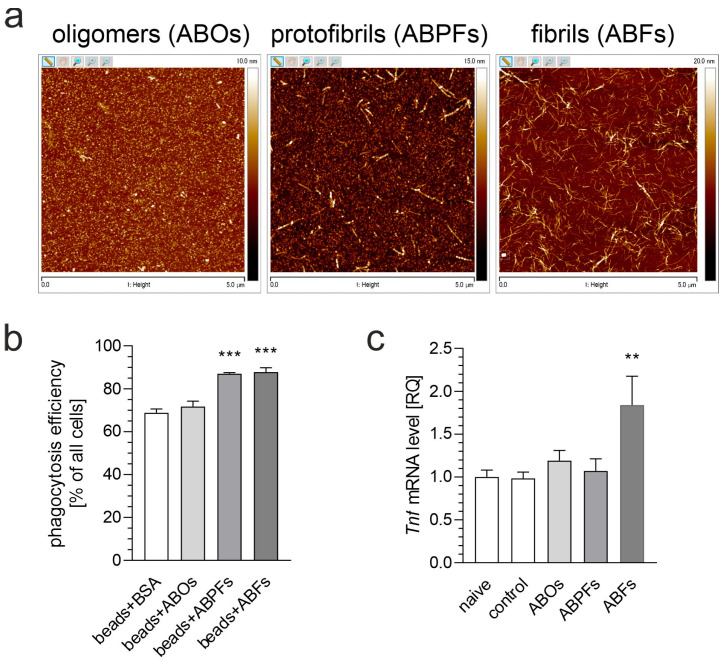
The effect of Aβ on BV2 cell activation. (**a**) Atomic force microscopy analysis of various Aβ preparations: oligomers (ABOs), protofibrils (ABPFs), and fibrils (ABFs). (**b**) The impact of 5 µM Aβ (ABOs, ABPFs, ABFs) on the phagocytic activity of BV2 cells. (**c**) The effect of 5 µM Aβ (ABOs, ABPFs, ABFs) on the mRNA level of the *Tnf* gene in BV2 cells after 6 h of incubation. Data are presented as mean ± SEM; n = 4 (**b**) and 6 (**c**). ** *p* < 0.01 and *** *p* < 0.001, compared to the respective control (ANOVA with Bonferroni post hoc test).

While microglial activation is commonly triggered by pro-inflammatory stimuli, such as LPS, it is also a central feature of neurodegenerative diseases associated with Aβ pathology. Therefore, in the following steps, we analyzed whether inhibiting BET proteins could protect neurons against the neurotoxic activity of microglia stimulated with Aβ. First, we examined how Aβ influences microglial activation (Figure 4) using two key indicators: phagocytosis and the expression of pro-inflammatory genes. Among the different forms of Aβ (oligomers, protofibrils, and fibrils), only Aβ fibrils (ABFs) effectively activated the microglial BV2 cells. The dose–response experiments showed that BV2 cell activation became detectable at an Aβ concentration of 5 µM, which was subsequently utilized in the following experiments. The exposure of the BV2 cells to ABFs at this concentration induced a significant upregulation of *Tnf* mRNA, yet this increase was less pronounced than the effect caused by LPS (compare Figure 2b).

**Figure 5:**
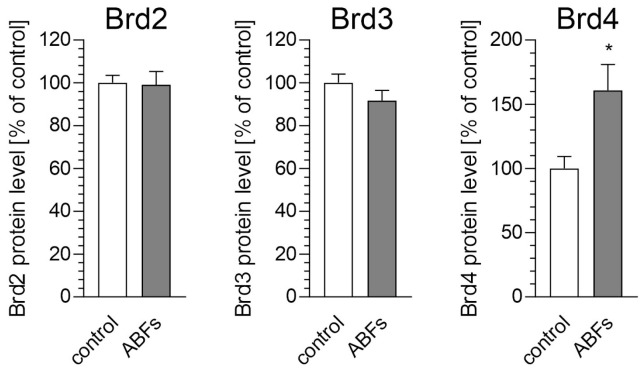
The effect of Aβ fibrils (ABFs) on BET protein levels in microglial BV2 cells. BV2 cells were incubated with ABFs (5 µM) for 6 h, and the levels of BET proteins were measured using ELISA assays. Data are presented as mean ± SEM; n = 3–4; * *p* < 0.05, compared to the control (Student’s *t*-test).

The ABFs treatment also led to the selective increase in Brd4 protein levels in the BV2 cells, with no significant alterations observed for Brd2 or Brd3 (Figure 5). This selective upregulation identifies Brd4 as a key mediator of microglial responses to amyloid pathology, underscoring its potential as a promising therapeutic target for neuroinflammation and Alzheimer’s disease (AD).

**Figure 6:**
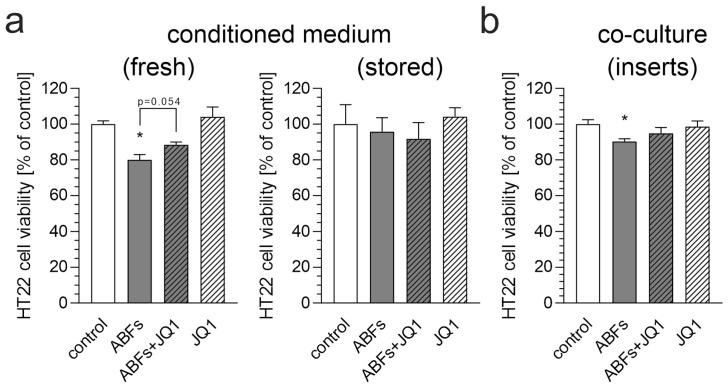
The effect of mediators released by ABFs-stimulated BV2 cells and/or JQ1 on the viability of HT22 cells. (**a**) BV2 cells were treated with JQ1 (50 nM) and/or after 30 min with ABFs (5 µM), and incubation was continued for 6 h. The conditioned medium was then transferred to murine neuronal HT22 cells, and after 24 h of incubation, cell viability was assessed using an MTT assay. (**b**) BV2 cells were treated with JQ1 (50 nM). After 30 min, ABFs (5 µM) were added, and incubation was continued. After 6 h, the medium was aspirated, and inserts with stimulated BV2 cells were transferred to HT22 cells. Incubation was then continued for an additional 24 h. Cell viability was assessed using an MTT assay. Data are presented as mean ± SEM; n = 4–5 (**a**), n = 8 (**b**); * *p* < 0.05, compared to the control group (ANOVA with Bonferroni post hoc test).

Conditioned media from the Aβ-stimulated BV2 cells caused a reduction in HT22 neuronal cell viability, highlighting the neurotoxic potential of microglia in response to fibrils (Figure 6a). However, this effect was observed only when the fresh medium was used. Storing the conditioned medium at −80 °C for a few days abolished its neurotoxic potential. Transwell inserts were utilized to establish a co-culture system. These inserts enable the physical separation of two cell types within a single well while facilitating media exchange between compartments. This design allows for the diffusion of soluble factors, such as cytokines, growth factors, and other molecules, from one compartment to the other, allowing for the investigation of their effects on cells without requiring direct cell-to-cell contact. Pre-treatment with JQ1 preserved the viability of the neuronal cells treated with a conditioned medium, but this effect was less pronounced in the co-culture studies (Figure 6a,b). This result suggests that BET proteins play a crucial role in mediating microglia-induced neurotoxicity, and their inhibition can mitigate the damaging effects of neuroinflammation on neurons. These findings support the idea that targeting BET proteins could be a promising neuroprotective approach for preventing or slowing neurodegenerative diseases linked to chronic neuroinflammation.

## 4. Discussion

Increasing evidence shows that the pathogenesis of AD is related to neuronal dysfunction and immune system dysregulation. Misfolded proteins activate microglial and astrocytic pattern recognition receptors, initiating an inflammatory response that accelerates disease progression. Genome-wide studies have associated risk genes with the regulation of external factors, such as systemic inflammation, which further exacerbates these processes [2,5,27,28]. Thus, modulating immune mechanisms and associated risk factors may offer promising therapeutic strategies for AD.

This study explored the role of BET family proteins, particularly Brd4, in regulating microglial activation and neuroinflammation, emphasizing the therapeutic potential of JQ1, a potent BET inhibitor. Our results indicate that Brd4 is a key regulator of microglial responses under pro-inflammatory conditions and that the inhibition of BET proteins can mitigate both the inflammatory response and neurotoxic effects in neurodegeneration models, including those induced by LPS and Aβ. These findings highlight the potential of BET inhibition as a novel therapeutic approach for neuro-inflammatory diseases, such as AD.

Our study demonstrated a significant upregulation of Brd4 in BV2 microglial cells after pro-inflammatory stimulation, both at the mRNA and protein levels. This finding is consistent with the existing literature, which suggests that Brd4 plays a central role in the transcriptional regulation of pro-inflammatory genes in glial cells, including microglia. Specifically, Brd4 is known to facilitate the transcription of inflammatory cytokines, such as TNF-α, IL-1β, and IL-6, by interacting with acetylated histones and coactivators, thus contributing to the inflammatory cascade [11,29]. Therefore, our data further support previous findings suggesting that Brd4 may be one of the primary proteins involved in regulating microglial activation under inflammatory conditions and possibly a specific therapeutic target in neuroinflammation-related disorders. Notably, the specific upregulation of Brd4 protein levels in the brain was also observed in our in vivo studies on LPS-induced systemic inflammation in mice (Matuszewska et al., submitted for publication). The significant increase observed after only 6 h of incubation may seem surprising. However, it is essential to note that both LPS and Aβ activate innate immune response mechanisms. This evolutionarily conserved process is optimized for rapid reactions to stimuli. The proteome’s highly dynamic response to LPS is well-documented, highlighting the potential for rapid and significant protein level changes even 60 min after stimulation [30]. Furthermore, considering Brd4’s role in inflammatory signaling, a rapid increase in its expression is both plausible and necessary if it plays a pivotal role in regulating genes involved in inflammation. Notably, other studies have documented the exclusive upregulation of Brd4 protein under stress conditions. For instance, murine heart tissue exhibited increased Brd4 mRNA and protein levels 12 h after intraperitoneal LPS administration [31]. Additionally, our previous in vivo study demonstrated an exclusive increase in Brd4 mRNA in the hippocampi of mice after systemic inflammation induced by LPS [32]. Data indicate that the elevated level of Brd4 persists for at least 24 h [33]. The discrepancy between the slightly increased mRNA level and the significantly increased Brd4 protein level in our experiment can be explained by differences in mRNA and protein stability. Consequently, even a moderate increase in mRNA levels can result in substantial protein accumulation. We hypothesize that this phenomenon is relevant to our study.

Additionally, our data showed that Aβ-induced microglial activation led to an increase in Brd4 protein expression, highlighting the crucial role of Brd4 in mediating the Aβ-related neuro-inflammatory process. This further supports the idea that Aβ influences the expression of this BET protein, thereby exacerbating the microglial response. This observation is consistent with previous studies demonstrating that Aβ triggers microglial activation through multiple mechanisms, including the modulation of chromatin remodeling factors, such as Brd4, which play a key role in the regulation of inflammatory gene expression [11].

Consistent with previous studies [29,34,35], our research demonstrated that BET inhibition significantly suppresses microglial activation and cytokine release. Additionally, our data showed that BET inhibition not only reduces inflammation but also promotes neuronal cell survival in pro-inflammatory environments. Thus, we extended the previous findings by demonstrating that BET proteins are key mediators of the toxic effects induced by microglia on neighboring neuronal cells. This observation might be particularly relevant for conditions associated with chronic neuroinflammation, such as AD, where microglial activation contributes to pathogenesis and neuronal death.

We also investigated the effects of Aβ, a key player in the pathomechanism of AD, on microglial activation. Our results showed that Aβ fibrils, but not Aβ oligomers or protofibrils, effectively induced microglial activation, as evidenced by enhanced phagocytosis and the elevated expression of pro-inflammatory cytokines, such as TNF. This finding is consistent with previous studies, which demonstrate that ABFs are the most potent activator of microglia, while soluble oligomers are less efficient at triggering inflammatory responses [36,37,38,39]. Some research indicates that ABFs induce microglial phagocytosis, while ABOs attenuate it [40]. Conversely, other studies report that ABOs elicit a stronger microglial inflammatory response than ABFs [41]. These disparate findings suggest that (i) ABOs and ABFs differentially activate microglia and (ii) the conformation of Aβ and experimental conditions must be rigorously controlled.

A key finding of this study was that pre-treatment with JQ1 conferred neuroprotection of HT22 neuronal cells exposed to Aβ-activated microglia. Conditioned medium from Aβ-stimulated BV2 cells significantly decreased the viability of HT22 cells. This neurotoxic effect was attenuated by pre-treatment with JQ1, indicating that BET inhibition can mitigate microglia-mediated neurotoxicity. This result is consistent with studies showing that BET inhibitors confer neuroprotection by attenuating microglial inflammatory responses following spinal cord injury in rats [29]. However, the consequences of the global inhibition of BET proteins require further investigation. A previous study on human neuronal-like cells demonstrated that pharmacological degradation and inhibition of BET proteins significantly increased Aβ levels [42]. On the other hand, other studies have shown that JQ1 and other BET inhibitors attenuate Aβ-induced neuroinflammation, preserve neuronal viability, and reduce neuronal loss in models of AD [35,43,44]. Moreover, several studies have demonstrated the beneficial effects of BET inhibitors on cognitive function. Prolonged administration of JQ1 improved cognition deficits in rat models of AD [45,46]. Additionally, JQ1 enhanced brain plasticity across various mouse models, including wild-type and APP-expressing mice, and effectively rescued hippocampal-dependent cognitive deficits observed in C9BAC mice, an animal model of frontotemporal dementia [47,48]. Despite its positive mitigating effects on inflammation and Tau phosphorylation, JQ1 failed to improve learning and memory deficits in 7-month-old 3×Tg-AD mice [44]. In contrast, another study demonstrated that prolonged administration of JQ1 had a negative impact on memory function in mice [49]. Moreover, administering JQ1 to young rats resulted in cognitive impairment in adulthood [50]. Finally, a human randomized controlled trial demonstrated that another BET inhibitor, Apabetalone (RVX-208), improved cognitive performance in patients aged 70 or older with a baseline MoCA score of 21 or less [51]. The neuroprotective effects of JQ1 in our study further support the notion that targeting Brd4 and other BET proteins can mitigate the neurotoxic effects of activated microglia in neurodegenerative diseases [47].

An intriguing finding was that fresh conditioned medium from stimulated microglia decreased neuronal cell viability, whereas medium stored at −80 °C for approximately a week did not exhibit this effect. Given that the secretome of stimulated BV2 cells comprises nearly 5000 proteins [52], speculation on this issue without supporting proteomic data is unreliable. Moreover, high-resolution quantitative proteomics analysis revealed fundamental differences in protein expression between human and murine microglia, as well as across various culture conditions [53]. Therefore, our results suggest that further studies on the human microglial cell system are necessary to resolve this puzzle. Besides proteins, lipid mediators, exosomes, RNAs, and ROS may be considered. Among the mediators released by activated microglia, ROS appear particularly vulnerable to long storage conditions due to their inherently unstable nature. ROS can still undergo reactions, albeit at a slower rate, even at −80 °C. However, cytokines also exhibit significant variability in stability during storage at −80 °C [54]. Notably, TNF-α, which has been demonstrated to be neurotoxic to HT22 cells [55], has been shown to degrade even under these storage conditions [54]. Exosomes are also vulnerable to −80 °C storage and freeze–thaw cycles [56], as prolonged storage under these conditions can alter their concentration, purity, and particle size.

Despite the inherent physiological differences between humans and mice, and the resulting limitations of using murine cells in this study, the significant homology (approximately 40%) between their genomes [57] suggests that the findings remain relevant. However, even with comparable gene control systems, RNA and protein expression can differ between mice and humans [53,58,59]. Further research in human cell systems is necessary to confirm our observations.

In summary, our study demonstrates that Brd4 plays a pivotal role in the inflammatory activation of microglia in response to LPS and ABFs. BET inhibition can effectively reduce both microglial inflammatory responses and neurotoxic effects. Given that chronic microglial activation is a central feature of many neurodegenerative diseases, our results provide strong evidence for the potential of BET inhibitors as a novel therapeutic approach for treating conditions characterized by neuroinflammation. Further preclinical and clinical studies are necessary to evaluate the efficacy of BET inhibitors in these diseases and to determine their therapeutic potential in human patients.

## 5. Conclusions

In conclusion, our study demonstrates that inhibition of the BET proteins attenuates microglial activation and neuronal damage in vitro, providing a potential therapeutic avenue for mitigating neuro-inflammatory degeneration in neurodegenerative diseases, such as Alzheimer’s disease.

## Data Availability

The data supporting the findings of this study are available on request from the corresponding author.

## Abbreviations

The following abbreviations are used in this manuscript:

Aβ	amyloid-β
ABOs	amyloid-β oligomers
ABFs	amyloid-β fibrils
ABPFs	amyloid-β protofibrils
AD	Alzheimer’s disease
AFM	atomic force microscopy
BET	bromodomain and extraterminal domain
CNS	central nervous system
DMSO	dimethyl sulfoxide
FMS	fluorescent microspheres
HFIP	hexafluoroisopropanol
LPS	lipopolysaccharide
PD	Parkinson’s disease
RQ	relative quantity
